# Obesity leads to underestimation of ventricular volumes and abnormal myocardial strain in repaired Tetralogy of Fallot as measured by cardiac MRI

**DOI:** 10.1186/1532-429X-16-S1-P112

**Published:** 2014-01-16

**Authors:** Scott A Simpson, Suzanne L Field, Neeru Kaushik, David Parra, Jonathan H Soslow

**Affiliations:** 1 Vanderbilt University Medical Center, Nashville, Tennessee, USA

## Background

Obesity is an increasing epidemic that has not spared children and adults with congenital heart disease. Patients with repaired tetralogy of Fallot with trans-annular patch (rTOF-TAP) have significant pulmonary insufficiency and right ventricular (RV) enlargement. Recommendations for pulmonary valve replacement (PVR) include body surface area (BSA)-indexed RV end diastolic volume (RVEDVi) >150 ml/m2 or indexed RV end systolic volume (RVESVi) >80 ml/m2, estimated by cardiac MRI (CMR). We hypothesized that: 1) overweight and obese patients with rTOF-TAP have underestimated indexed ventricular volumes when compared to volumes indexed to ideal BSA and 2) these patients have altered parameters of cardiac function compared to weight appropriate patients.

## Methods

Retrospective review of 86 patients from 2009-2013 with rTOF-TAP who underwent CMR. Mean age 20.1 years (± 10.9). Patients assigned weight categories by Center for Disease Control guidelines for children and adults based on body mass index (BMI): appropriate weight (n = 51), overweight (n = 23), and obese (n = 12). CMR analysis included: 1) RV volumes and RV ejection fraction (RVEF); 2) Left ventricular (LV) volumes and LVEF; 3) Peak circumferential LV strain (ε_cc_) using HARP analysis of myocardial tagged images. Overweight and obese patients assigned ideal BSAs to recalculate indexed RV volumes. Mann-Whitney U was used to compare continuous variables between groups.

## Results

**Figure 1:**
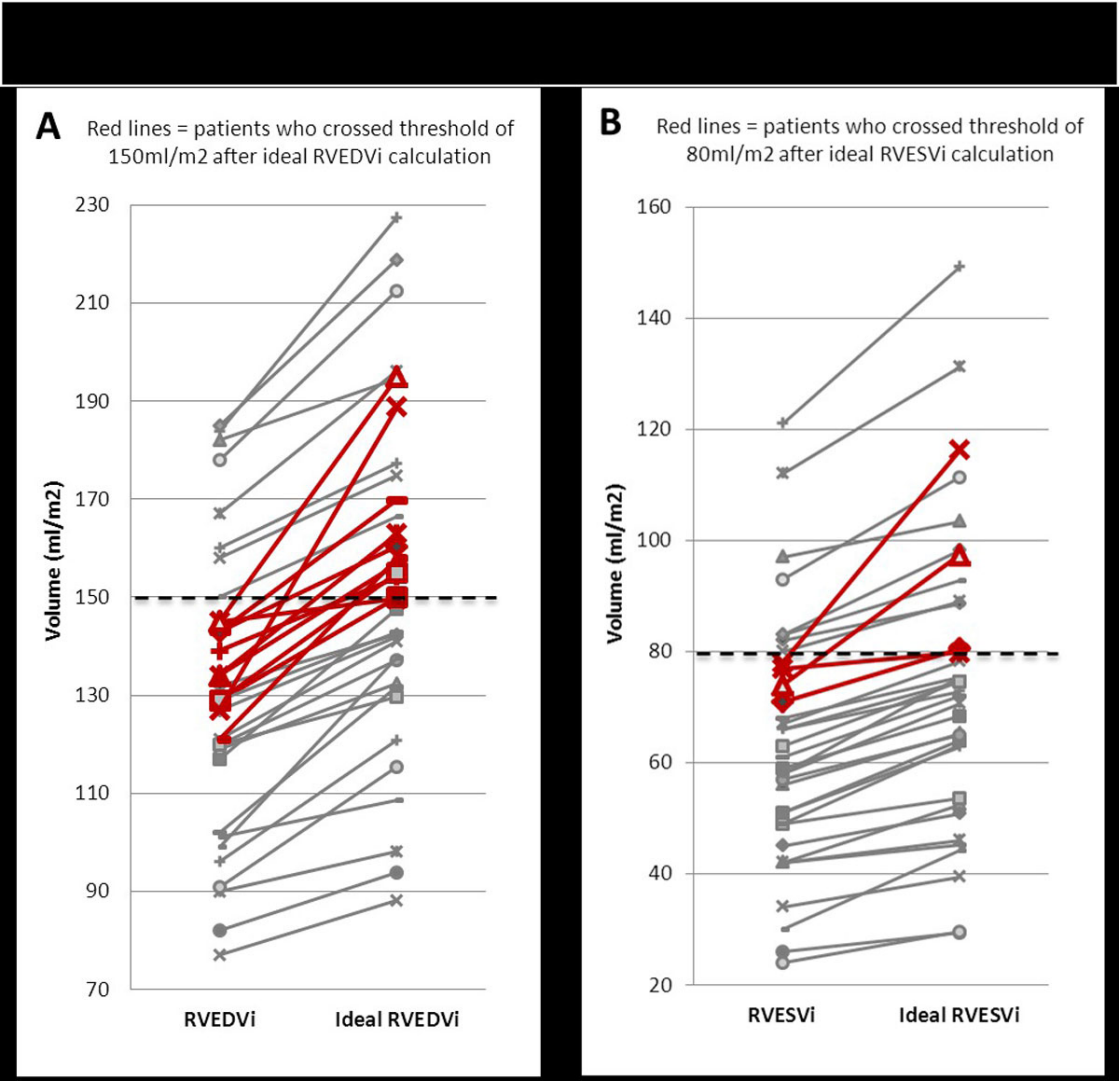


**Figure 2:**
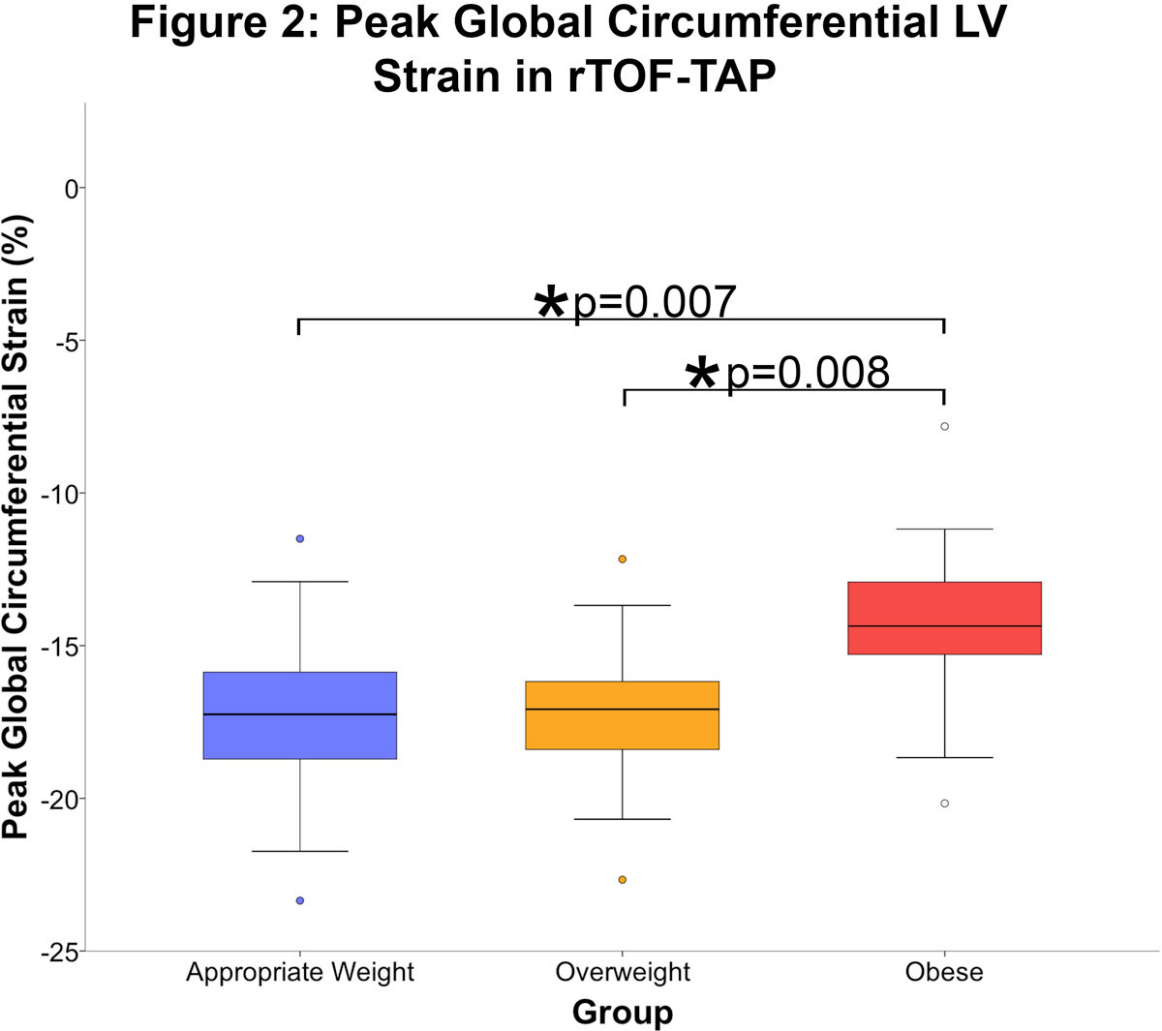
**Peak global cicumferential LV strain in rTOF-TAP**.

Mean BMI in appropriate weight 19.8 kg/m2, overweight 26.4 kg/m2, obese 33.5 kg/m2. Obese and overweight patients had significantly larger absolute RVEDV and LVEDV compared to weight appropriate patients (RV mean 234 ml vs. 200 ml, p = 0.014; LV mean 113 ml vs. 92 ml, p = 0.002). No significant difference in RVEF and LVEF among groups. When RV volumes were corrected for ideal BSA, 11 (31%) additional overweight and obese patients met standard criteria for PVR referral (Figure 1); mean change RVEDVi 22.9 ml/m2 (range 4.8-62 ml/m2), mean change RVESVi 11.3 ml/m2 (range 2.9-38.4 ml/m2). There was a statistically significant difference between ε_cc _in appropriate weight (-17.3%) and obese (-14.3%) patients, (-2.94%, 95%CI{-5.2,-0.6} p = 0.007) (Figure 2); segmental analysis between appropriate weight and obese patients demonstrated decreased ε_cc _in anteroseptal, inferior, and inferolateral segments (p < 0.001, p = 0.003, p = 0.016, respectively). No significant differences in ε_cc _noted between appropriate weight and overweight patients.

## Conclusions

Increased BMI leads to the underestimation of indexed RV volumes, possibly affecting timing of PVR. This underestimation should be corrected using ideal BSA. Decreased LV ε_cc _has not been previously reported in obese patients with rTOF-TAP. Although clinical implications of abnormal ε_cc _are unclear, these patients may be at higher risk for early LV dysfunction. Further studies on ε_cc _in this patient population are recommended.

## Funding

Funded in part by NIH T32HL 105334.

